# Clinical and biological features of neuroblastic tumors: A comparison of neuroblastoma and ganglioneuroblastoma

**DOI:** 10.18632/oncotarget.17146

**Published:** 2017-04-17

**Authors:** Wen-Guang He, Yu Yan, Wen Tang, Rong Cai, Gang Ren

**Affiliations:** ^1^ Department of Radiology, Xinhua Hospital, Shanghai Jiaotong University Medical School, Shanghai 200092, China; ^2^ Department of Radiotherapy, Ruijin Hospital, Shanghai Jiaotong University Medical School, Shanghai 200025, China; ^3^ Department of Pathology, Xinhua Hospital, Shanghai Jiaotong University Medical School, Shanghai 200092, China

**Keywords:** neuroblastoma, ganglioneuroblastoma, clinical and biological features

## Abstract

Neuroblastoma (NB), ganglioneuroblastoma intermixed (GNBi) and ganglioneuroblastoma nodular (GNBn) are neuroblastic tumors that present with a wide range of symptoms and variable prognoses. We retrospectively reviewed the pretreatment clinical (age, sex and tumor stage) and biological (MYCN amplification; and levels of lactate dehydrogenase, ferritin and neuron-specific enolase) characteristics of 279 patients who were diagnosed with pathologically confirmed NB and GNB from January 2005 to December 2015. The median age at diagnosis increased with grade of differentiation (NB: 28.9 months; GNBn: 38.4 months; GNBi: 47.5 months; p < 0.01). NB patients were more frequently diagnosed with adrenal tumors and had a higher prevalence of abnormal serum ferritin at the time of diagnosis (60.0% vs. 40.0% vs. 12.0%, P<0.001), NSE (96.0% vs. 93.0% vs. 81.0%, P=0.013) when compared with GNBn and GNBi patients. The prevalence rates of disseminated tumors and MYCN amplified tumors were lower in the GNBi group than in the GNBn and NB groups (13.0% vs. 25.0% vs. 44.0%, P=0.002; 0 vs. 14.0% vs. 26.0%, P=0.032, respectively). The overall survival (OS) of patients with GNB was significantly better than that of patients with NB (GNBi: 100%, GNBn: 74.5±11.4%, NB: 50.8±4.5%, respectively; P<0.01). Our study revealed that both NB and GNB have a wide range of presentations, and clinicians should be aware of both typical and atypical symptoms and signs. Children with GNB (especially GNBi) were more likely to present favorable prognostic factors than their NB counterparts, which consequently lead to better outcomes and longer survival for these patients.

## INTRODUCTION

Neuroblastoma (NB); ganglioneuroblastoma, intermixed (GNBi); ganglioneuroma (GN); and ganglioneuroblastoma, nodular (GNBn) represent a spectrum of neuroblastic tumors arising from primitive sympathetic ganglion cells; additionally, these solid extra-cranial tumors are frequently identified in children [1, 2]. These tumors vary in their relative proportions of Schwann cells and neuroblasts and can be distinguished by their degree of cellular maturation and differentiation. NB is malignant in nature; GN is considered to be a benign tumor, while GNB has intermediate malignant potential according to International Neuroblastoma Pathology Classification (INPC) system [3]. Analyses conducted by De Bernardi B et al. suggested that GNBi behaves similarly to GN and can be considered to be at the benign end of the peripheral neuroblastic tumor (pNT) spectrum [4]. Furthermore, an analysis performed by the International Neuroblastoma Risk Group (INRG) indicated that the prognosis of and response to therapy in GNB patients with the nodular subtype were significantly more unfavorable than those of GNBi patients [5]. A recent study found that GNB with nodular tumors represent a small group of pNT and are associated with heterogenous outcomes [6]. Therefore, although NB and nodular GNB are both considered to be malignant tumors, their clinical and biological features might differ. In this study, we retrospectively evaluated the clinical (age, sex and tumor stage) and biological (MYCN amplification; levels of lactate dehydrogenase, ferritin, vanillylmandelic and neuron-specific enolase) features of NB, GNBn and GNBi to provide more evidence to inform clinical diagnoses and therapeutic approaches.

## RESULTS

### Comparison of the clinical and biological features of NB and GNB

The clinical and biological characteristics of 279 neuroblastic tumors are illustrated in Table 1. Of the participants, 228 were NB patients; 20 were confirmed to be GNBn patients, while 31 were GNBi patients. No gender differences were identified between the three groups. NB patients were diagnosed at a younger median age (28.9 vs 38.4 vs 47.5 months). Patients with NB were more likely to have adrenal primary tumors (55.0% vs. 20.0% vs. 42.0%, P=0.005) and less likely to have a thoracic tumors (9.0% vs. 30 .0% vs.26.0%, P=0.005) than were GNBn and GNBi patients. Consistent with the more favorable outcomes observed in this group of patients, GNB patients, especially those with the intermixed subtype of tumor, more frequently had normal serum ferritin levels (88.0% vs. 60.0% vs.40.0%, P<0.001), LDH (15.0% vs. 10.0% vs. 3.0%, P=0.025) and NSE (19.0% vs. 7.0% vs. 4.0%, P=0.013) when compared with NB patients. Twelve (26.0%) of the 47 tumors analyzed in patients with NB were MYCN amplified, while only 1 case of GNBn had MYCN amplification. The prevalence of MYCN amplified tumors was higher in the NB group than in the GNB groups (P=0.032). In addition, NB patients were more likely to have disseminated disease compared with GNBn and GNBi patients (44.0% vs. 25.0% vs. 13.0%, p=.0.002), who most frequently had localized disease (75.0%, 87.0% respectively).

**Table 1:** Comparison of characteristics for NB and GNB patients at diagnosis

Variables	NB (N, %)	GNBn (N, %)	GNBi (N, %)	P value
**Age, months**				<0.01
Median	28.9	38.4	47.5	
**Gender**				0.637
Male	150(66)	12(60)	18(58)	
Female	78(24)	8(40)	13(42)	
**Primary Tumor Sites**				0.005*
Adrenal	125(55)	4(20)	13(42)	
Retroperitoneum	66(29)	10(50)	4(13)	
Neck	3(1)	0	2(6)	
Thorax	21(9)	6(30)	8(26)	
Pelvis	6(3)	0	2(6)	
Other	7(3)	0	2(6)	
**24-hr urine VMA**				<0.001
Normal	82(41)	11(79)	21(81)	
Abnormal	118(59)	3(21)	5(19)	
**LDH (U/L)**				0.025*
Normal	6(3)	1(10)	4(15)	
Abnormal	180(97)	9(90)	22(85)	
**Ferritin (ng/ml)**				<0.001
Normal	58(40)	6(60)	21(88)	
Abnormal	86(60)	4(40)	3(12)	
**NSE (ng/ml)**				0.013*
Normal	5(4)	1(7)	5(19)	
Abnormal	137(96)	14(93)	21(81)	
**MYCN**				0 .032*
Non-amplified	35(74)	6(86)	19(100)	
Amplified	12(26)	1(14)	0	
**Tumor stage**				0.002
Localized(L1, L2)	128(56)	15(75)	27(87)	
Disseminated (M, MS)	100(44)	5(25)	4(13)	

LDH, lactate dehydrogenase; NSE, neuron-specific enolase; VMA, vanillylmandelic acid.

*Fisher's exact test.

### Clinical and biological features of neuroblastic tumors according to demographic characteristics

We compared the clinical and biological characteristics of neuroblastic tumors with respect to sex and age (Table 2). Of the 279 patients, 94 were diagnosed before the age of 18 months, while 185 patients were older than 18 months at the time of diagnosis. The results of the statistical analysis revealed a relationship between tumor stage and patient age. Over the study period, 86 (46%) patients who were older than 18 months of age presented with M-stage disease. Conversely, 23 (25%) of the pediatric patients younger than 18 months old presented to our center with high-risk M stage disease (46.0% vs. 25.0%, P<0.01). The prevalence of elevated levels of serum ferritin was higher in patients older than 18 months of age compared with patients younger than 18 months of age (59.0% vs. 38.0%, P=0.008). However, there were no obvious correlations between patient age and other biological features. No gender differences were identified in the clinical and biological features assessed in this study.

**Table 2:** Clinical and biologic features of NB and GNB with respect to the patient age and gender

Variables	Age			Gender		
	< 18m	> 18m	P	Male	Female	P
	N, %	N, %		N, %	N, %	
**24-hr urine VMA**			0.957			0.704
Normal	37(47)	76(47)		73(46)	40(49)	
Abnormal	42(53)	85(53)		85(54)	42(51)	
**LDH (U/L)**			0.738			0.336
Normal	4(6)	7(5)		5(4)	6(8)	
Abnormal	63(94)	148(95)		135(96)	76(92)	
**Ferritin (ng/mL)**			0.008			0.140
Normal	36(62)	49(41)		47(41)	33(42)	
Abnormal	22(38)	71(59)		68(59)	30(58)	
**NSE (ng/mL)**			1.0			0.502
Normal	4(6)	7(6)		5(4)	5(7)	
Abnormal	58(94)	114(94)		110(96)	62(93)	
**MYCN**			0.918			0.524
Non-amplified	24(83)	36(82)		38(8)	22(9)	
Amplified	5(17)	8(18)		9(92)	3(91)	
**Tumor stage**			<0.001			0.084
Localized(L1, L2)	71(75)	99(54)		101(56)	67(67)	
Disseminated(M, MS)	23(25)	86(46)		79(44)	32(33)	

### Analysis of clinical presentation at initial diagnosis

**Figure 1:**
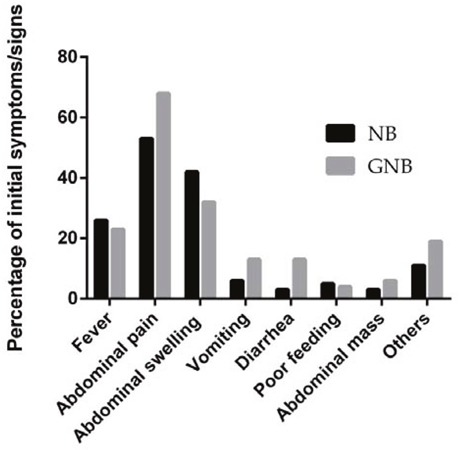
Presenting symptoms and clinical signs of NB and GNB

The presenting symptoms and clinical signs of NB and GNB among patients whose primary tumor site was abdominal at the time of diagnosis are presented in Figure 1. The most common symptoms were abdominal pain (53.0%), followed by abdominal swelling (42.0%) and fever (26.0%). Similarly, the most common presentation identified in GNB patients was abdominal pain (68.0%), while 42% children had abdominal swelling, and 26% of patients had fever as their initial symptoms at the time of hospital admission. Vomiting, diarrhea and poor feeding were also identified in a small proportion of patients. A small number of patients presented with clinical signs such as palpable abdominal masses with or without abdominal pain. Others presented with limb weakness, bone pain, or enlarged cervical masses, which were related to metastases.

### NB and GNB tumor stage based on primary tumor site

Patients were categorized into groups by tumor origin (abdominal and extra-abdominal origin), and tumor stage at diagnosis was determined according to the primary tumor site (Table 3). Within the NB group, 191 tumors were of abdominal origin and 52% of tumors were localized (L1 or L2), while 48% of tumors were stage M or Ms. Of the remaining 37 extra-abdominal NB patients, 28 (68.0%) had localized disease, while 32% of patients had disseminated disease. The prevalence of disseminated tumors was higher in the abdominal group than in the extra-abdominal group (48.0% vs. 32.0%, P=0.009). In both the GNBn and GNBi group, there was no obvious correlation between primary tumor site and stage.

**Table 3:** Tumor stage of neurobalstic tumors with respect to tumor origin

Tumor Stage	Abdomen (N, %)	Extra-abdomen(N, %)	P
**NB**			0.009
Localized(L1, L2)	100 (52)	28(68)	
Disseminated(M, MS)	91 (48)	9(32)	
**GNBn**			1.0*
Localized(L1, L2)	10(71)	5(83)	
Disseminated(M, MS)	4(29)	1(17)	
**GNBi**			0.607*
Localized(L1, L2)	14(82)	13(93)	
Disseminated(M, MS)	3 (18)	1(7)	

*Fisher exact test.

### Metastatic sites based on primary tumor site

We evaluated whether metastatic site differed according to primary tumor site (abdominal and extra-abdominal) (Table 4). The most common site of metastasis was the bone marrow (68%), followed by the bone (44%). There was an obvious relationship between bone marrow invasion and the primary site of the tumor. Patients with extra-abdominal metastatic tumors were less likely to have bone marrow metastasis than were patients with tumors of abdominal origin (15.0% vs. 75.0%, P<0.001). However, there were no significant differences identified in brain, liver, lung, skin, distant lymph node metastasis or bone invasion at diagnosis between patients with abdominal and extra-abdominal tumors.

**Table 4:** Metastases sites of patients with disseminated tumors by tumor site

Metastatic Site	All (N, %)	Abdominal (N, %)	Extra-abdominal (N, %)	P
Bone marrow	74 (68)	72(75)	2(15)	< 0.001
Bone	48(44)	39(41)	9(69)	0.051
Brain	2 (2)	2(2)	0	1.0
Liver	9 (8)	9(9)	0	1.0
Lung	1 (1)	1(1)	0	1.0
Skin	2 (2)	1(1)	1(8)	0.196
Distant lymph nodes	9 (8)	7(7)	2(15)	0.232

### Distribution of bone metastasis sites

**Figure 2:**
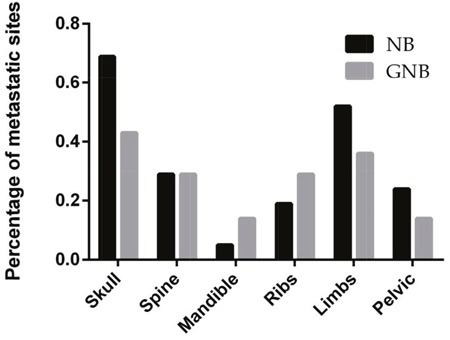
Distribution by percentage of bone metastatic sites in children with NB and GNB

Bone metastases involved the skull, limbs, pelvis, spine, ribs, scapula or mandible (Figure 2). We found that the skull was the most common site of bone metastasis, which accounted for 69% of bone metastases in patients with NB and 43% of bone metastases in patients with GNB. The second most common site of metastasis was the limbs. Multiple bone metastasis sites were identified in 12 NB patients and 3 GNB patients.

### Survival

**Figure 3:**
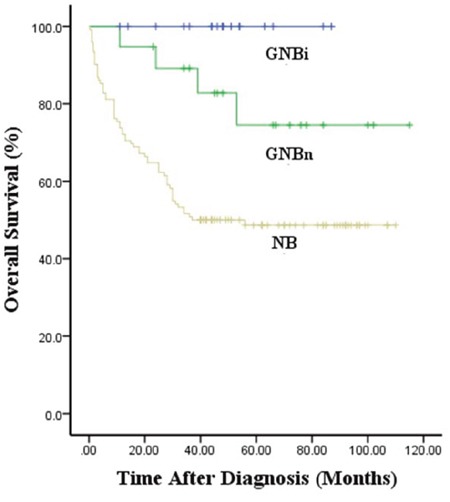
Kaplan–Meier survival curves in patients with NB, GNBn and GNBi

**Figure 4:**
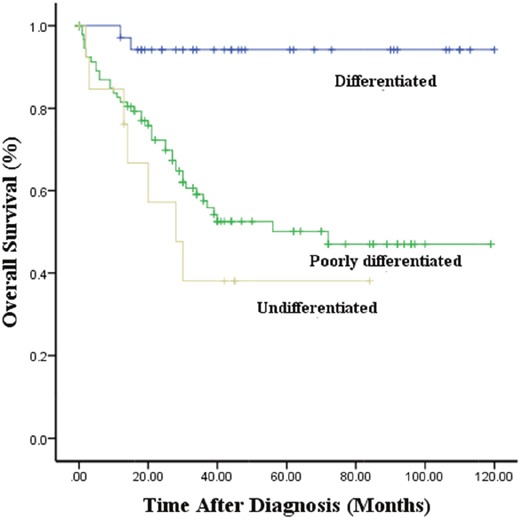
Kaplan–Meier survival curves in NB with different grade of differentiation

The median survival times were 43.8, 51.0 and 64.5 months for patients with NB, GNBn and GNBi, respectively. All 31 patients with GNBi were alive at the time of analysis. The 5-year overall survival rates in patients with NB, GNBn and GNBi were 50.8±4.5%, 74.5±11.4%, 100%, respectively (P<0.01) (Figure 3). Among the NB patients, the 5-year overall survival rates in patients with differentiated, poorly differentiated and undifferentiated tumors were 82.3±5.6%, 50.1±5.9%, 38.1±1.5%, respectively (p<0.001) (Figure 4).

## DISCUSSION

Neuroblastoma is the most common extracranial solid tumor in childhood and displays a broad spectrum of clinical behavior [7]. Ganglioneuroblastoma represents a histological subgroup of neuroblastic tumors with intermediate malignancy potential that categorized as nodular (GNBn) and intermixed (GNBi) [3, 8]. Thus far, histology has been the only way to differentiate between these tumor-types, as it has proven difficult for differentiation to be achieved in imaging studies [9, 10]. Due to its variable differentiation, it is possible that NB, GNBn and GNBi have different clinical and biological features. Therefore, this study was designed to analyze differences in the clinical and biological features of NB and GNB.

Patient age, tumor stage, histology, tumor grade, MYCN oncogene status, chromosome 11q status, and DNA ploidy have been reported to be the most clinically relevant prognostic factors in previous studies [5, 11–14]. Our study showed that patients with NB were more likely to display unfavorable prognostic factors when compared with GNB patients, while in the comparison of patients with confirmed GNBi and patients with GNBn, GNBi patients more frequently demonstrated favorable factors, as most GNBi tumors were localized and no MYCN amplification was found in GNBi patients. It has been suggested that MYCN amplification is strongly associated with rapid tumor progression and poor prognosis [15]. These findings indicated that NB displayed more aggressive behaviors, while GNBi often presented favorable behaviors. In our study, we also found patients with GNBi to have a higher survival rates than patients with GNBn. This is consistent with published data suggesting that patients with GNBn may have very poor prognosis [5]. As previously reported, GNBi and GN behaved in a similarly benign manner [4]. None of the GNBi patients died during follow-up. This indicated that patients with GNBi should not undergo aggressive treatment. In our institution, the 5-year survival rate identified in patients with NB was 50.8%, which remained dissatisfactory despite the application of multiple therapies. Considering the variable differentiation identified in patients with NB, we found that the 5-year survival rates of patients with poorly differentiated and undifferentiated tumors were worse than that of patients with differentiated tumors.

Previous studies have reported that NB patients who were diagnosed at >18 months of age had poorer prognosis than those diagnosed at a younger age [16]. In the overall cohort, we found that patients with neuroblastic tumors who were older than 18 months were more likely to have disseminated tumors. However, in terms of biological features, both of the groups shared similar results. In addition, previous studies have indicated that children who had cervical, thoracic, and pelvic primary neuroblastomas were more likely to have favorable clinical and biological features than were children with tumors of abdominal origin [17–18]. Patients with advanced-stage tumors were more likely to have abdominal NB, a finding that is consistent with previous studies. However, no relationship was identified between tumor stage and the primary tumor site in GNBn and GNBi. Whether extra-abdominal origin is an independent favorable prognostic factor in NB patients requires further analysis.

NB and GNB patients had similar clinical presentations. Abdominal pain and swelling are common manifestations at the first clinical visit. Other associated manifestations included vomiting, diarrhea, poor and metastasis-related disease. Approximately 60% of NB patients have been found to present with evidence of metastases at the time of initial diagnosis [19]. These patients may present with symptoms related to metastatic disease such as bone pain, limping, or an enlarged cervical mass. Thus, the clinical presentations of neuroblastic tumors are complicated, and clinicians should not only be aware of classic clinical presentations but also atypical manifestations, especially those caused by disseminated tumors; this may help to ensure that a prompt diagnosis be made and effective therapeutic protocols can be implemented to increase survival rates and minimize irreversible damage.

Prior studies have suggested that NB often metastasized to the bone marrow (70.5%), bone (55.7%), lymph nodes (30.9%), and liver (29.6%) [20, 21], while tumor metastasis to the lung or brain were rare [22–25]. Based on the hypothesis that tumors from different sites demonstrate varying clinical features, we sought to determine whether metastatic sites differ according to primary tumor site. The results showed that patients with tumors of abdominal origin were more likely to have bone marrow metastasis. With the exception of the bone marrow, the study conducted by Kieuhoa et al. suggested that patients with extra-abdominal tumors had lower rates of bone metastasis, while patients with abdominal metastatic tumors were more likely to have liver metastasis than were patients with other primary tumor sites [13]. However, in our study, no associations were found between tumor and metastatic site.

The bone was the second most common metastatic site in patients with NB, and its detection may be important for tumor staging and risk stratification, as it is for other metastatic diseases. Here, we found that the skull was the most common metastatic site in both NB and GNB, which was consistent with the results of previous studies [26]. A proportion of the patients in our study had multiple bone metastasis sites. Multiple bone metastases may prove to be both a diagnostic and therapeutic challenge, and patients with suspected or confirmed NB or GNB should have a whole-body bone scan performed via multiple imaging modalities.

There were several limitations to our study. First, only a small number of patients underwent amplification MYCN examination. Additionally, detection of DNA ploidy and allelic deletions on chromosomes 1p and 11q and gains of 17q was not performed in our hospital. Another limitation was the small sample size of our study; thus, further studies including larger sample size and genetic data should be performed.

## CONCLUSIONS

In this study, we described the clinical and biological characteristics of children with confirmed NB and GNB, and the results revealed that both types of tumors have a wide range of presentations. Although the tumors shared similar clinical manifestations, patients with GNBi were more likely to demonstrate favorable prognostic factors than were patients with GNBn and NB. The outcomes identifed in patients with NB were worse than those identified in patients with GNBn and GNBi. Clinicians should be aware of the various clinical presentations, and the use of multiple imaging and clinical tests is essential to both clinical diagnosis and therapy.

## MATERIALS AND METHODS

### Patients

This retrospective study was approved by the Institutional Ethics Committee of Xinhua Hospital, and the requirement of informed consent was waived. Data from total of 279 patients younger than 18 years of age with pathologically confirmed NB or GNB who were diagnosed in Xinhua Hospital, which is affiliated with Shanghai Jiao Tong University School of Medicine, between 2005 and 2015 were retrospectively reviewed. Two hundred and twenty-eight cases of NB (57 differentiated, 117 poorly differentiated and 14 undifferentiated) and 51 cases of GNB (20 GNB, nodular; 31 GNB, intermixed) were identified. The tumors were staged according to the International Neuroblastoma Risk Group Staging System (INRGSS) [27, 28], a preoperative staging system published by Tom Monclair et al. in 2009 that is based on tumor image-defined risk factors rather than the extent of a surgical resection. Clinical information (age, sex, site of the primary, presentation, stage) and biological information (neuron-specific enolase (NSE), vanillylmandelic acid (VMA), lactate dehydrogenase (LDH), ferritin and MYCN gene status) were collected at the time of the initial diagnosis. The original tumor and any disseminated lesions were evaluated using computerized tomography (CT), magnetic resonance imaging (MRI), technetium-99 m bone scans, bilateral bone marrow aspirates and biopsy specimens.

### Statistical methods

Descriptive data are presented in terms of absolute frequencies, and percentages are presented for qualitative data. For between group comparisons, categorical variables were examined using the Pearson's chi-square test or the Fisher's exact test. Time-to-event for OS was defined as time from enrollment to death or time of last contact if the patient was alive. Survival was analyzed using the Kaplan-Meier method, and comparisons between groups were performed using the log-rank test. All statistical analyses were completed using SPSS for Windows, version 21.0 (SPSS, Chicago, IL, United States). For all analyses, P values less than 0.05 were considered statistically significant.
